# Supplementary material for: Acetylcholine and Royal Jelly Fatty Acid Combinations as Potential Dry Eye Treatment Components in Mice
Source: Nutrients. 2021 Jul 24;13(8):2536. doi: 10.3390/nu13082536 (PMC8399524; doi:10.3390/nu13082536)
Supplement: Supplementary file 1 [file nutrients-13-02536-s001.zip › nutrients-1284924-supplementary.pdf]

Supplementary Information for

Acetylcholine and royal jelly fatty acid combinations as potential dry eye treatment components in mice.

Masayuki Yamaga [1]† & Toshihiro Imada [2]†, Hiroko Tani [1], Shigeru Nakamura [2], Ayanori Yamaki [1], Kazuo Tsubota [2,3] *

[1] Institute for Bee Products and Health Science, Yamada Bee Company, Inc., Okayama, 708-0393, Japan; my1636@yamada-bee.com (M.Y.); ht0807@yamada-bee.com (H.T.); ay1255@yamada-bee.com (A.Y.)

[2] Department of Ophthalmology, Keio University School of Medicine, Tokyo, 160-8582, Japan; imada_toshihiro@keio.jp (T.I.); s-nakamura.a5@keio.jp (S.N.); tsubota@z3.keio.jp (K.T.)

[3] Tsubota Laboratory, Inc., Shinjuku-ku, Tokyo, 160-0016, Japan; tsubota@z3.keio.jp (K.T.)

† These authors contributed equally to this work.

* Correspondence: Email: tsubota@z3.keio.jp Phone number: +81-6384-2866

Table of contents

• **Experimental section**

• **Scheme S1.** Synthesis of (*E*, *R*)-11, 12-dihydroxy-2-dodecenoic acid.

• **Figure S1.** ^{1}H NMR spectrum of (*E*, *R*)-11, 12-dihydroxy-2-dodecenoic acid.

• **Figure S2.** ^{13}C NMR spectrum of (*E*, *R*)-11, 12-dihydroxy-2-dodecenoic acid.

• **Table S1.** Validation data of the analytical methods for acetylcholine and RJ fatty acids**.**

Experimental section

General Procedure

Optical rotation was measured using a HORIBA SEPA-500 digital polarimeter. IR spectra were recorded by the attenuated total reflection method using ZnSe prism on a JASCO FTIR-4100 spectrophotometer. ^{1}H and ^{13}C NMR spectra were recorded at JEOL ECA600 spectrometers. Chemical shifts were referenced to a residual signal of CD_3OD (δ_H 3.30) or the solvent signal (δ_C 49.0). HRMS were recorded on a Thermo-Fisher Scientific Orbitrap Q Exactive focus mass spectrometer.

(*E*, *R*)-11, 12-Dihydroxy-2-dodecenoic acid

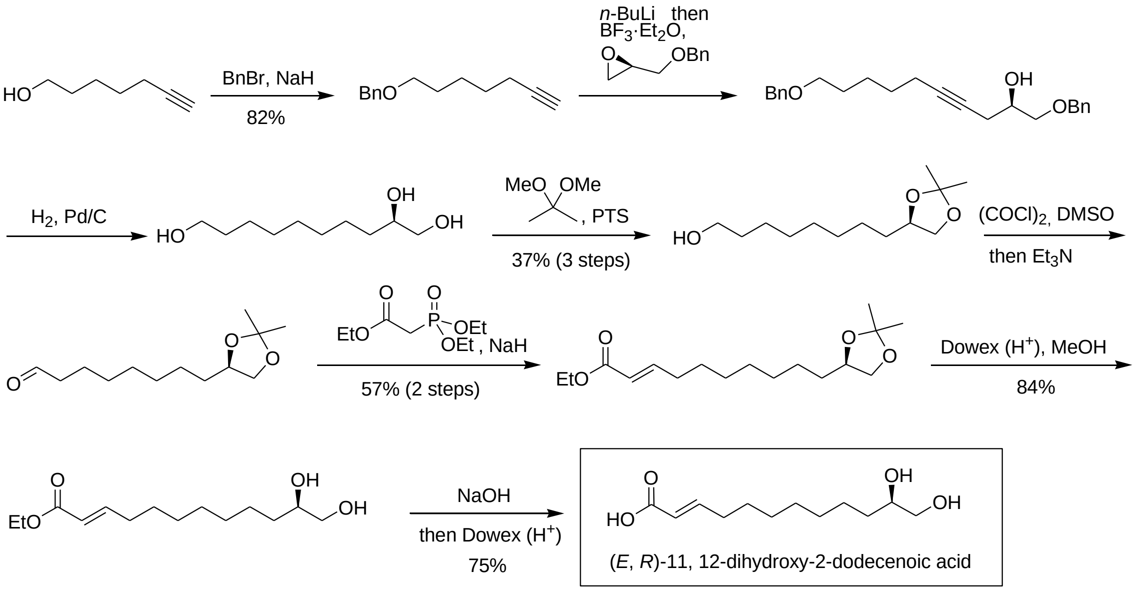

Scheme S1. Synthesis of (*E*, *R*)-11, 12-dihydroxy-2-dodecenoic acid.

The title compound was prepared according to the procedures described in the patent (US2019/0230967 A1 [26]) and Scheme S1, and confirmed the structure by NMR and mass spectra.

$[\alpha]_D^{24}$ = +5.1 (*c* 0.55, methanol); IR (ZnSe) 3522, 3203, 2913, 2841, 1710, 1652, 1449, 1290, 1173, 1014 cm^{-1}; ^{1}H NMR (CD_3OD, 600 MHz): δ 6.94 (dt, *J*=15.6, 7.2 Hz, 1H), 5.78 (dt, *J*=15.6, 1.2 Hz, 1H), 3.55 (m, 1H), 3.46 (dd, *J*=11.4, 4.8 Hz, 1H), 3.40 (dd, *J*=11.4, 6.6 Hz, 1H), 2.21 (ddt, 7.2, 1.2, 7.8 Hz, 2H), 1.52-1.36 (m, 12H); ^{13}C NMR (CD_3OD, 150 MHz) δ 170.8, 151.3, 122.5, 73.3, 67.4, 34.3, 33.1, 30.3, 30.1, 29.9, 29.2; negative-ion ESIMS: *m/z* 229 $[M-H]^-$; HRMS (ESI) calcd for $C_{12}H_{22}O_4$ $[M-H]^-$ 229.1445, found 229.1444.

Figure S1. ^{1}H NMR spectrum of (*E*, *R*)-11, 12-dihydroxy-2-dodecenoic acid (CD_3OD, 600 MHz)

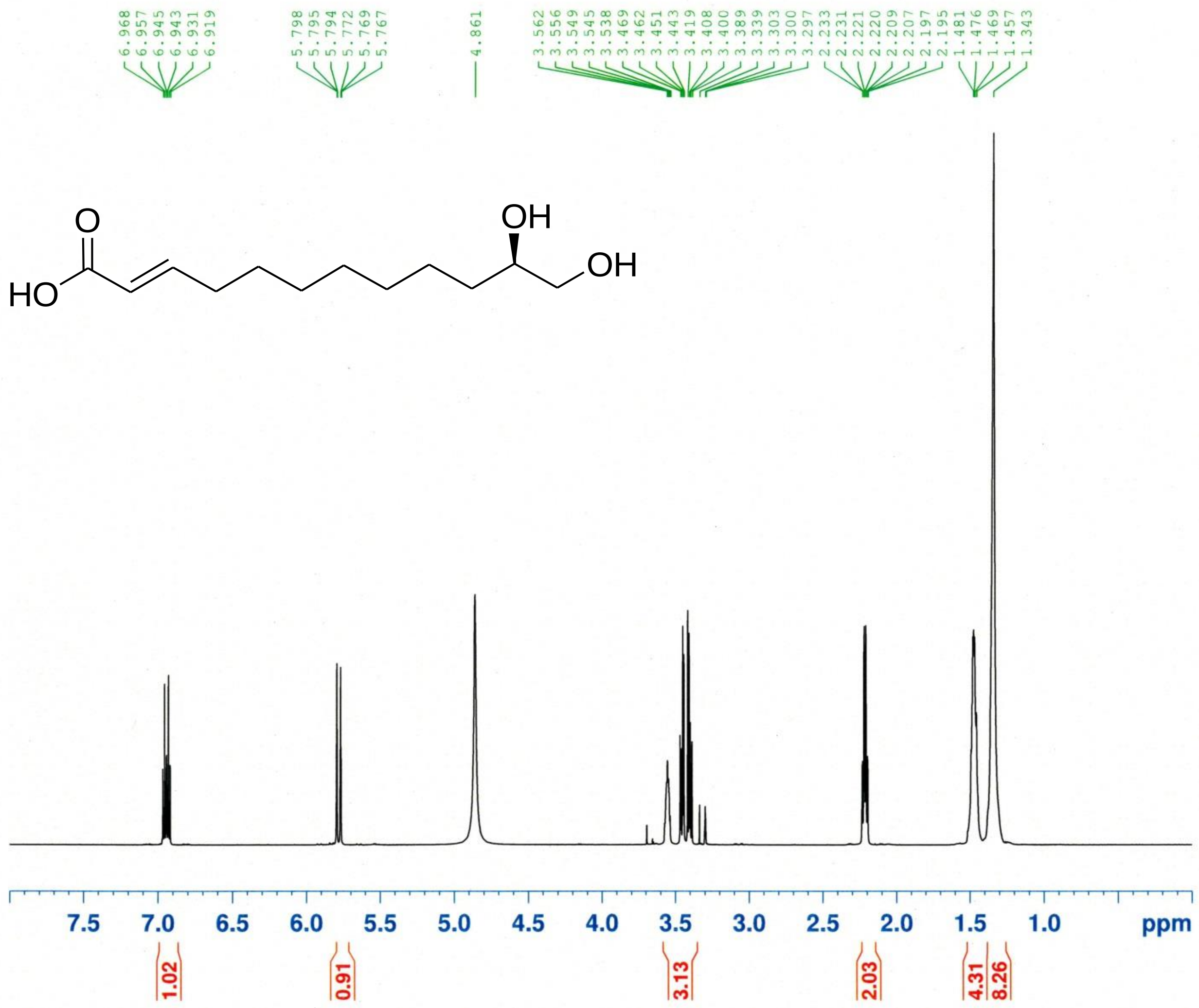

Figure S2. ^{13}C NMR spectrum of (*E*, *R*)-11, 12-dihydroxy-2-dodecenoic acid (CD_3OD, 150 MHz)

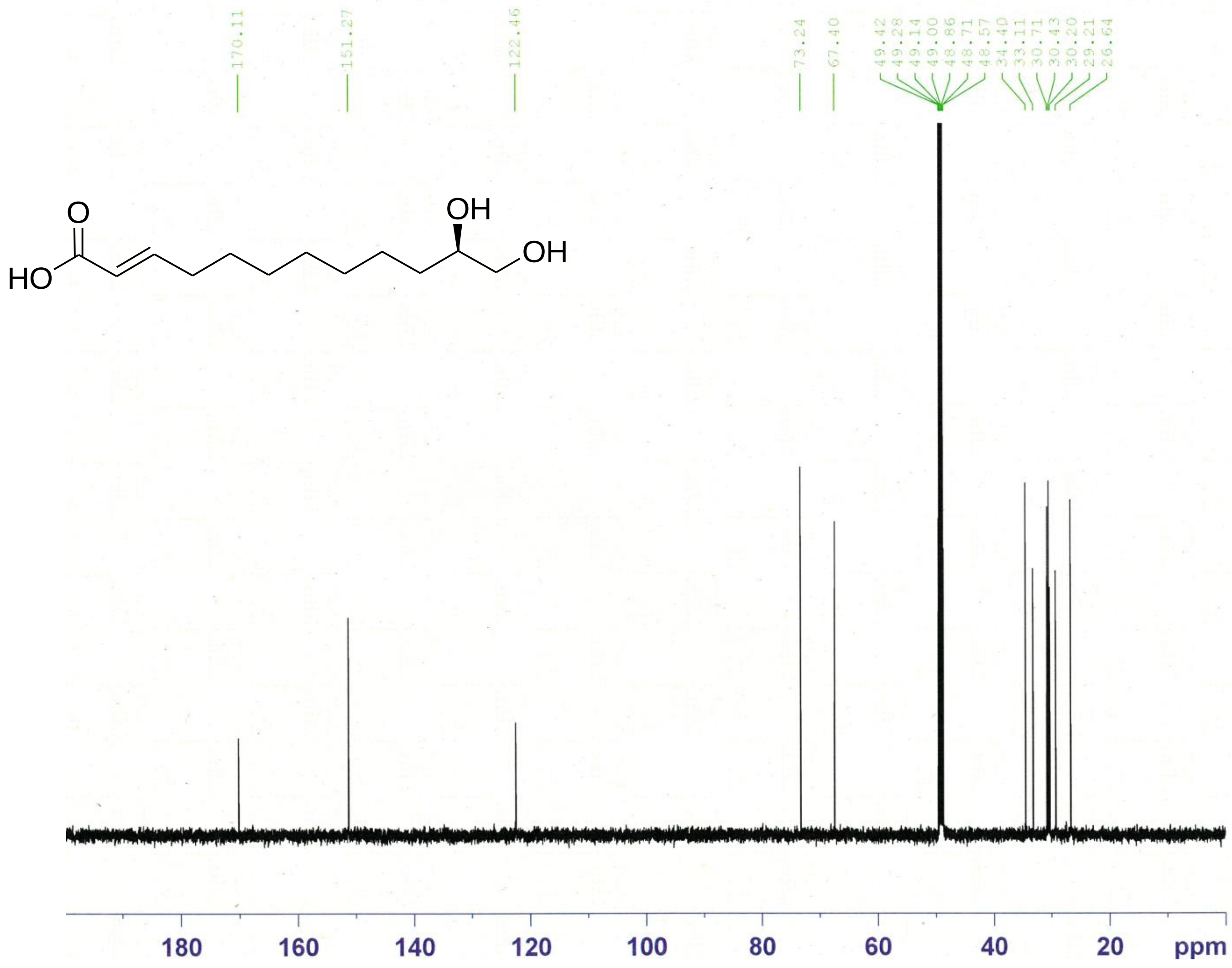

Table S1. Validation data of the analytical methods for acetylcholine and RJ fatty acids.

Compounds	Accuracy (Recovery) (%)	Repeatability (RSD %)	Reproducibility (RSD %)	LOD (S/N=3) (ng/mL)	LOQ (S/N = 10) (ng/mL)	Range (ng/mL)	R^2
Acetylcholine (ACh)	106.35	3.81	4.46	0.13	0.43	1-1000	0.999
8-hydroxyoctanoic acid (8HOA)	107.58	5.03	5.25	0.34	1.14	1-1000	0.999
(*R*)-3,10-dihydroxydecanoic acid (3,10DDA)	108.89	4.20	4.79	0.40	1.33	1-1000	0.999
10-hydroxydecanoic acid (10HDAA)	101.15	3.14	3.30	0.48	1.58	1-1000	0.999
(*E*)-9,10-dihydroxy-2-decenoic acid (9,10D2DA)	106.15	10.16	11.37	0.60	2.01	1-1000	0.999
(*E*)-10-hydroxy-2-decenoic acid (10H2DA)	113.34	4.57	4.69	0.66	2.20	1-1000	0.999
(*E*)-2-decenedioic acid (2DA)	110.11	4.26	4.80	0.35	1.17	1-1000	0.999
Sebacic acid (SA)	107.14	9.90	9.93	3.82	12.72	1-1000	0.999
(*E, R*)-11,12-dihydroxy-2-dodecenoic acid (11,12D2DA)	107.82	2.37	3.35	0.39	1.30	1-1000	0.999
12-hydroxydodecanoic acid (12HDA)	109.31	11.92	13.55	0.46	1.54	1-1000	0.999
